# Reconstruction and the Sunday Fund

**Published:** 1919-06-21

**Authors:** 


### Hospital Sunday, June 22, 1919.

### *SPECIAL HOSPITAL SUNDAY FUND SECTION.*

### RECONSTRUCTION AND THE SUNDAY FUND.

The unfortunate clergy, the worst-paid professional body in the country, are rather weary of begging, and there are few more urgent problems of reconstruction than that of church finance. But to beg for outside causes is one thing, to preach on Hospital Sunday is another. As we make a practice of reminding both clergy and congregations at this season, Hospital Sunday was the creation of one of the clergy themselves. The idea, on which the whole swarm of annual " days " has been modelled, was invented by the late Canon Miller of Birmingham in 1859. The idea was not merely to raise money, but to devote one Sunday in the year to teach the lesson of personal service, to remind the well of their obligation to the sick, and to show them that there was no better use for their leisure than to devote themselves to that kind of public work which lies nearest to all doors—namely, hospital maintenance and management. Since the health of parochial life depends upon the participation of intelligent laymen in its organisation, since the chief business of a minister of religion is in the church rather than outside it, the welfare of every parish depends upon the gospel of personal service no less than the welfare of hospitals. But hospitals provide the best illustration for this teaching, partly because no parable is more popular than that of the Good Samaritan, and mainly because the appeal of pain and distress is more instantly felt than any other. Englishmen pride themselves on doing voluntarily those things which other nations have to be compelled to do. We pride ourselves on the voluntary principle. We are jealous of State interference. We dislike red tape. Reconstruction, therefore, means to us the setting up of those voluntary organisations which have been suspended during the War in the stress of its emergency. In the case of the Hospital Sunday Fund, which many suppose to be languishing, reconstruction means the revival of the voluntary principle, the restatement of the doctrine of personal service; and this can be urged sincerely only to preachers who believe in it. Whether the clergy believe in Hospital Sunday as they once did is open to legitimate doubt, because, in the course of the year, they are expected to plead for so many causes—from missions to church expenses—that their own cause, Hospital Sunday, is apt to be merged in the mass and not given that precedence which is its due.

An appeal merely for money is not convincing from a pulpit. The address has to be a sincere insistence on a principle, among the results of which is the arousing of generosity. In the world of business, in which most members of a typical congregation are engaged, personal service comes to mean competitive effort, usually for a purpose in which the higher activities are not called into play. It is a relief, therefore, to such workers to be offered a field of public work in which they can be disinterested, in which they are helping to spend money rather than to make it, and for which the end in view is merely the best that can be done for the patient. This perhaps is the attraction which leads some of the best brains in the city to devote their leisure to the management of voluntary hospitals, for which much of the work, as well as the funds, are given voluntarily. The art of life consists in making the most of opportunities near at hand, and, consequently, those who wish to give personal service, something of their own health and strength to the hospitals, should turn their attention to their local hospital. Though every voluntary hospital is open to the public, many people fail to make their acquaintance and to know of the work which is going on almost at their doors. But the impulse of the War, which attracted many voluntary workers to all kinds of activity, including hospital work, should not be lost. Indeed, it is common to hear of men and women, who at the call of the War left their own homes, now unwilling to give up the active life which they began in an emergency. They have learnt the satisfaction of personal service, the interest which it adds to life; and thus after making the experiment no one wishes to abandon it permanently. It is the business of the Hospital Sunday Fund movement to keep this impulse alive now that the War is over, as it fostered it before the War began, and to remind every congregation that personal service is an opportunity which is constant, however much it may be emphasised in a crisis.

The needs of reconstruction can be met only in two ways, by the multiplication of authorities and enactments, or by voluntary agencies. But the latter, which alone can save us from regimentation on the Prussian plan, can only spring from the public spirit of men and women who regard themselves as trustees for their own health, and wish to spend it for an impersonal cause, one that alone can call forth their highest energies. The Sunday Fund movement exists to emphasise every year the motive of such people, to announce the principle by which they act, and to make the call to which they have responded. It is the centre of a movement greater than itself, and is the witness to the rewards of those who have yielded to their generous impulses.

